# Risk factors for increased postoperative drainage of calcaneal fractures after open reduction and internal fixation: An observational study

**DOI:** 10.1097/MD.0000000000011818

**Published:** 2018-08-10

**Authors:** Zitao Zhang, Zhen Wang, Yan Zhang, Xusheng Qiu, Yixin Chen

**Affiliations:** a Department of Orthopedics, Drum Tower Hospital Clinical College of Nanjing Medical University; b Department of Orthopedics, Drum Tower Hospital Clinical College of Nanjing University, Nanjing; c Department of Intensive Care Unit, The First People's Hospital of Changshu, Changshu, P.R. China.

**Keywords:** calcaneal fractures, open reduction and internal fixation, postoperative drainage, risk factors

## Abstract

Sufficient drainage is very important for preventing wound complications after open reduction and internal fixation (ORIF) of calcaneal fractures. However, the drainage amount varies among patients. The objective of this study was to identify factors associated with increased postoperative drainage after ORIF of calcaneal fractures.

A retrospective study including 87 patients with 92 calcaneal fractures in our hospital was performed. Patients were divided into 2 groups based on whether they had increased drainage, which was defined as a total drainage of ≥340 mL (50th percentile). We gathered the following data on each patient: age; sex; smoking history; body mass index (BMI); American Society of Anesthesiologists (ASA) classification; fracture type; the time from injury to surgery; operative time; bone grafting; preoperative labs including prothrombin time (PT), activated partial thromboplastin time (APTT), hematocrit, and D-dimer level; and histories for hypertension, diabetes, and heart disease. Univariate analysis and multivariate logistic regression analysis were used to analyze the risk factors associated with increased drainage.

Total drainage ranged from 105 to 1185 mL, and the average drainage for this cohort was 393.6 ± 232.4 mL (mean ± standard deviation). 57.6% (n = 53) of patients had increased drainage. Smoking history, Sanders type, operative time, and bone grafting were significantly associated with increased drainage on univariate analysis. Multivariate logistic regression analysis then demonstrated that active smoking and higher Sanders type were independent risk factors for increased drainage.

Patients with calcaneal fractures who smoked or had a higher level of Sanders type had a higher risk of increased postoperative drainage. Therefore, we suggest that active precautions be taken for these patients to reduce the rate of postoperative wound complications.

## 1 Introduction

Calcaneal fractures are one of the most frequently encountered fractures of the hindfoot^[1,2]^ and have been estimated to account for approximately 1% to 2% of all fractures.^[3]^ High-energy trauma is the main cause and often results in displaced intra-articular calcaneal fractures. Currently, open reduction and internal plate fixation (ORIF) is an effective surgical procedure used to restore calcaneal height and the mechanical axis of the hindfoot and subtalar joint; it improves patient quality of life and functioning.^[4]^

However, due to factors such as specific anatomical features, inadequate postoperative management, and insufficient postoperative drainage, calcaneal fractures are frequently associated with postoperative wound complications including infection, hematoma, and epidermal necrosis.^[5]^ Some patients must even undergo surgical debridement, hardware removal, or skin grafting. Recent studies suggest sufficient postoperative drainage can not only decrease hematoma formation but also stimulate granulation tissue growth, thereby promoting wound healing.^[6,7]^ However, the drainage amount after calcaneal fracture ORIF varies among patients. Thus, screening for risk factors for increased postoperative drainage is needed to help ensure sufficient drainage.

Until now, no studies have focused on risk factors for increased postoperative drainage of calcaneal fractures. Hence, a retrospective study was designed to obtain useful clinical information that can then be used to help reduce the risk of postoperative wound complications.

## 2 Patients and methods

All participants provided written informed consent for the experimental procedures, which were in accordance with the Declaration of Helsinki and were approved by the Medical Ethics Committee of Drum Tower Hospital Clinical College of Nanjing Medical University.

We conducted a retrospective analysis of 125 patients diagnosed with calcaneal fractures in our institution from January 2014 to December 2016. For inclusion in the study, the patients had to be no <18 years of age and be diagnosed with a closed calcaneal fracture. Exclusion criteria for patients were as follows: lack of consent; incomplete information; age younger than 18 years; open calcaneal fracture; the presence of an ankle fracture on the same side; a Sanders type I calcaneal fracture; the presence of diabetes or a vascular disease of the lower limbs; obvious soft-tissue infection or skin disease; the use of conservative treatment or percutaneous fixation. Thirty-eight patients were excluded: 8 patients lacking consent, 5 patients with incomplete information, 2 patients <18 years, 4 patients with open calcaneal fractures, 3 patients with an ankle fracture on the same side, 9 patients with a Sanders type I calcaneal fracture, 5 patients treated conservatively, and 2 patients treated using a percutaneous technique. Finally, 87 patients with 92 calcaneal fractures were enrolled in this study.

Once ORIF procedures were identified, we gathered the following data on each patient: age; sex; smoking history; body mass index (BMI); American Society of Anesthesiologists (ASA) classification; fracture type; the time from injury to surgery; operative time; bone grafting (yes or no); preoperative labs including prothrombin time (PT), activated partial thromboplastin time (APTT), hematocrit, and D-dimer level; and histories for hypertension, diabetes, and heart disease. The Sanders classification system was used to determine the fracture type by x-ray, computerized tomography (CT) scan, and 3-dimensional reconstruction.^[8]^ Type I fractures were excluded because they were nondisplaced and were treated conservatively.

All patients were admitted to the hospital for foot elevation. Surgery was performed when the swelling had diminished, so that skin wrinkles were visible. Patients received general anesthesia and were placed in the contralateral decubitus position on the injured side. A pneumatic tourniquet was applied to the injured limb and inflated to 240 mmHg. The calcaneal fracture was then treated using a lateral L-shaped skin incision with plate internal fixation performed by the same surgeon. Autogenous bone grafts, allogenous bone grafts, or artificial bone grafts were used for fractures with severe compression to fill bone defects. To decrease wound complications, each patient had a closed suction drain (8 F) placed before the incision was closed with a 2-layered closure.

Drainage was defined as the total output from the time of surgery until the time of drain removal and was measured in milliliters. Postoperatively, the drain was removed when the drainage was <30 mL over the preceding 24 hours so no significant drainage was missed.^[9]^ The primary outcome measure was increased drainage, and we defined this binary variable as positive when the drainage was greater than or equal to the 50th percentile of drainage for this cohort.^[10]^

Continuous variables are presented as means ± standard deviations (SDs), and categorical variables as absolute numbers and proportions. We used the Student *t* test to analyze continuous data. The Kruskal–Wallis test was used for ordered categorical data or non-normally distributed data. Categorical data were tested using the chi-square test or Fisher exact test, as appropriate. We conducted a multivariable logistic regression analysis to further analyze the risk factors for increased postoperative drainage after ORIF of calcaneal fractures. A prediction algorithm with reliable odds ratios (ORs) and 95% confidence intervals (95% CIs) was achieved.^[11]^ All statistical analyses were performed using SPSS 19.0 system software (SPSS Inc., Chicago, IL). Only variables for which the *P*-value was <.05 in the final model were considered significant. All *P*-values were two-sided.

## 3 Results

### 3.1 General data

In all, 87 patients with 92 calcaneal fractures meeting the inclusive criteria were treated between January 2014 and December 2016. There were 69 men and 18 women, with a mean age of 45.4 ± 11.6 years and a mean BMI of 24.1 ± 3.1 kg/m^2^. There were 5 patients with bilateral calcaneal fractures and 82 patients with unilateral calcaneal fractures. The main cause of injury was falling, accounting for 73.9% of cases (n = 68). According to imaging data, Sanders type III–IV calcaneal fractures accounted for 45.7% (n = 42) of cases. Among the patients, 41.3% (n = 38) were active smokers at admission. Before being injured, 13 patients had been diagnosed with hypertension, 3 patients with diabetes, and only 2 patients with heart disease. The mean drainage was 393.6 ± 232.4 mL (range, 105–1185 mL). Overall, 57.6% (n = 53) had increased drainage (drainage ≥50th percentile, or ≥340 mL). Furthermore, none of the patients with bilateral calcaneal fractures had increased drainage.

### 3.2 Analysis of the potential risk factors

Several potential risk factors for increased drainage were compared and analyzed (Table 1). Bivariate analysis showed that age; sex; hypertension history; diabetes history; heart disease history; BMI; ASA classification; preoperative PT, APTT, hematocrit and D-dimer; and time from injury to surgery were not significant risk factors for increased drainage. However, smoking history (*P* = .034), Sanders fracture types III–IV (*P* = .006), longer operative time (*P* = .045), and bone graft use (*P* = .049) were significantly higher in those with increased drainage. The above 4 variables were then entered into multivariable logistic regression analysis (Table 2), and only smoking history (OR = 2.68, 95% CI = 1.03–6.98, *P* = .044) and Sanders fracture types III–IV (OR = 3.58, 95% CI = 1.40–9.12, *P* = .008) remained statistically significant for increased drainage.

**Table 1:**
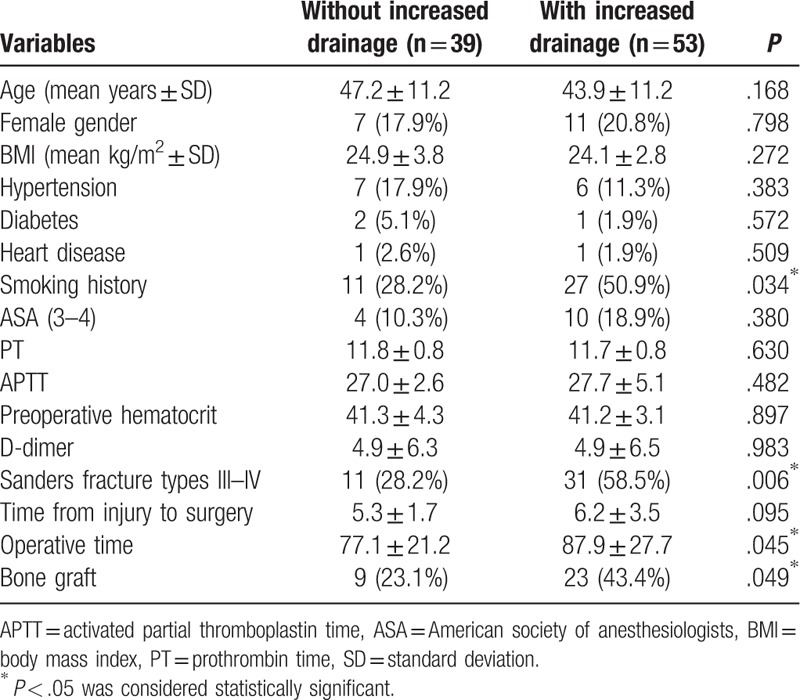
Clinical characteristics of the patients with or without increased drainage.

**Table 2:**
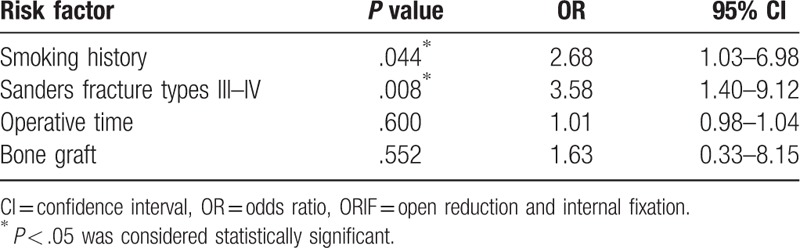
Multivariate regression analysis of possible risk factors for increased drainage in patients with a calcaneal fracture surgery with ORIF.

## 4 Discussion

In our study, smoking history and Sanders fracture types III–IV were found to be independently associated with increased drainage. Age; sex; BMI; ASA classification; time from injury to surgery; operative time; bone grafting; preoperative PT, APTT, hematocrit and D-dimer; and history of hypertension, diabetes, and heart disease were found not to be significant risk factors for increased drainage after ORIF.

ORIF has been proven to be an effective treatment method for improving outcomes and reducing morbidity for patients with calcaneal fractures. However, the reported rate of wound complications remains too high.^[12–14]^ Because wound complications usually have profound negative effects on patient recovery, many studies have identified risk factors for postoperative wound complications after ORIF of calcaneal fractures. Abidi et al^[12]^ demonstrated single-layered closure, high BMI, extended time between injury and surgery, and smoking as risk factors; whereas, Soni et al^[15]^ found that all the deep infections that occurred in his cohort were in smokers. Recently, there has been a focus on drainage after calcaneal fracture surgery because sufficient postoperative drainage can not only decrease hematoma formation, but also stimulate granulation tissue growth.^[6,9]^ Zhang et al^[6]^ reported that no drainage obviously increases the risk of postoperative wound complications. Meanwhile Backes et al^[9]^ found that the use of a closed suction drain was effective for avoiding wound complications. However, the drainage amount after surgery varies among patients. Therefore, identifying risk factors for increased postoperative drainage could help inform surgeons of the need for active precautions to decrease wound complications. No relevant data have been sought until now, and ours is the first study to seek to identify risk factors for increased postoperative drainage in patients having undergone calcaneal fracture ORIF.

It is generally acknowledged that smoking has many detrimental effects for patients undergoing orthopedic surgery. Smoking is a modifiable risk factor for decreased wound healing, increased infections, and impaired fracture union.^[16–19]^ Moller et al^[20]^ has reported that a history of smoking has long been associated with poor wound healing, which contributes to increased drain output after elective orthopedic surgery. In this study, patients with history of smoking had a 2.68 relative risk of increased drainage compared with those without a history of smoking (*P* = .044). Research suggests that smoking increases drainage by decreasing cutaneous blood flow, tissue oxygenation, and the inflammatory healing response.^[21,22]^ Furthermore, preoperative smoking cessation has been shown to decrease postoperative wound-related complications including hematoma after orthopedic surgery.^[23]^ Therefore, based on our results, it is recommend that smoking-cessation should be recommended before ORIF.

Our analysis also identified greater fracture severity (Sanders type III–IV) as another patient-related factor associated with increased drainage after ORIF of calcaneal fractures. We found that the OR for increased postoperative drainage for those with Sanders type III–IV patients was 3.58 (*P* = .008). Comparably, Zhang et al^[6]^ collected 10 observational studies involving 1559 patients with 1651 calcaneal fractures and confirmed that fracture severity (OR = 3.31) had a positive correlation with postoperative wound complications. Greater fracture severity usually results from more severe trauma, with increased swelling and greater destruction of the surrounding soft tissues, which can result in disruption of the soft tissue microcirculation.^[24]^ In addition, more complex fractures are theoretically associated with longer surgical times, larger incisions, more extensive exposure, greater blood loss and more prolonged anesthesia, factors which are likely to increase drainage.^[25]^

It is usually accepted that more severe and complex fractures require longer operative times and more frequent use of bone grafting. Univariate analysis showed longer operative times (*P* = .045) and more bone graft use (*P* = .049) in those with increased drainage; however, multivariate analysis revealed that these factors were not related to increased drainage. Koski et al^[26]^ reported that longer surgical time was statistically associated with incision complications. Bajammal et al^[27]^ suggested that bone grafting prevented hematoma by filling the bone defect. On the contrary, Wu et al^[28]^ demonstrated that bone grafting made no significant difference. Due to the small number of cases and various complications, we think that larger studies are required to establish the true relationship, if any, between these 2 factors and postoperative drainage.

This is the first study to identify risk factors for increased postoperative drainage in patients with calcaneal fractures with the goal of allowing surgeons to use this data to take active precautions to decrease wound complications. However, there were several limitations of our study. Firstly, it was retrospective in design and included a relatively small number of cases. Indeed, the clinical importance of our results would be greater with larger sample sizes. Secondly, some drainage might have been missed because any residual volume was not recorded after drain removal. However, the drains were only removed if there was minimal output, so significant drainage should not have been missed. Finally, there is no set guideline on what amount of drainage results in an increased occurrence of complications.

## 5 Conclusion

A history of smoking and Sanders type III–IV fractures were found to be independent risk factors for increased postoperative drainage in patients after calcaneal fracture ORIF. We propose that patients who have these risk factors be treated with active precautions to avoid wound complications.

## Author contributions

**Conceptualization:** Zitao Zhang, Zhen Wang.

**Data curation:** Zhen Wang, Yan Zhang.

**Funding acquisition:** Zitao Zhang.

**Investigation:** Zitao Zhang.

**Methodology:** Zitao Zhang.

**Project administration:** Xusheng Qiu.

**Resources:** Zhen Wang.

**Supervision:** Yixin Chen.

**Validation:** Yixin Chen.

**Writing – original draft:** Zhen Wang.

**Writing – review and editing:** Zitao Zhang.
